# Effect of estimated glomerular filtration rate and fluid balance on clinical course and outcomes of children admitted with severe dengue

**DOI:** 10.1186/cc14078

**Published:** 2014-12-03

**Authors:** N Vijayakumar, S Kandasamy, T Sangaralingam, VV Varadarajan, N Krishnamoorthi

**Affiliations:** 1 Department of Pediatrics, Mehta Children's Hospital, Chennai, India; 2 Department of Pediatric Intensive Care, Mehta Children's Hospital, Chennai, India; 3 Department of Academics and Research, Mehta Children's Hospital, Chennai, India

## Introduction

Dengue fever is one of the most important seasonal epidemics in Asia Case fatality rates vary from 1 to 5% [1]. Mortality from severe dengue may range from 26% in DHF to as high as 47% in DSS [2,3]. The pathogenesis of shock in dengue fever (DF) is centered on increased capillary permeability in the critical phase leading to hypovolemia and shock in severe dengue. There have been multiple studies that compare fluid regimens in the management of dengue [4,5]. These studies do not assess the child's renal function and ability to handle the fluid loads. GFR <60 ml/minute indicates a significant decrease in the renal functioning and there are no pediatric studies that examine their association with in-hospital stay and outcomes in children with severe dengue. With this introduction we formulated this study protocol to examine that association. The objectives were to measure the estimated glomerular filtration rate (eGFR) at admission and fluid balance in the first 36 hours of ICU stay and assess their effect on disease course and outcomes in severe dengue.

## Methods

This was designed as a retrospective descriptive study in a tertiary-level pediatric ICU in South India. Case records of all children fulfilling the WHO case definition of severe dengue were included, those who received intravenous fluid for less than 12 hours were excluded. Primary parameters measured included fluid balance in the first 36 hours measured every 12 hours, durations of oxygen requirement, mechanical ventilation, ICU stay and total hospital stay. Outcomes measured were death and survival.

## Results

Twenty-six children were enrolled, 14 boys and 12 girls. The median duration of ICU stay was 60 hours, and that of hospital stay 109 hours. eGFR was less than 60 ml/minute in six patients (83.3% expired and 16.7% survived). eGFR, measured by modified Schwartz's formula, at the time of admission correlated inversely with requirement of oxygen therapy and mechanical ventilation (*P *< 0.05) and fluid balance in the first 36 hours. Positive fluid balance (FO > 15%) in the first 36 hours was significantly higher in children who expired (*P *= 0.011). eGFR <90 ml/minute at admission had 100% sensitivity and 79% specificity to predict the possible occurrence of fluid overload >15% (area under curve = 0.882).

## Conclusion

Fluid balance in the first 36 hours had a significant positive correlation with mortality and negative correlation with eGFR. Children with admission eGFR <90 ml/minute may require restrictive fluid therapy to improve survival.
